# Gene Expression Aberrations in Alcohol-Associated Hepatocellular Carcinoma

**DOI:** 10.3390/ijms251910558

**Published:** 2024-09-30

**Authors:** Andreja Petrović, Paula Štancl, Paula Gršković, Suzana Hančić, Rosa Karlić, Slavko Gašparov, Petra Korać

**Affiliations:** 1 Division of Molecular Biology, Department of Biology, Faculty of Science, University of Zagreb, 10000 Zagreb, Croatia; petrovic.andrea@gmail.com (A.P.); pstancl@bioinfo.hr (P.Š.); rosa@bioinfo.hr (R.K.); 2 Institute of Clinical Pathology and Cytology, Merkur University Hospital, 10000 Zagreb, Croatia; suzana.hancic@kb-merkur.hr (S.H.); slavko.gasparov@kb-merkur.hr (S.G.); 3 Department of Pathology, Medical School Zagreb, University of Zagreb, 10000 Zagreb, Croatia

**Keywords:** *NTF-3*, *MYBL1*, hepatocellular carcinoma, alcohol etiology, miRNA-221

## Abstract

Hepatocellular carcinoma (HCC) is the most prevalent primary liver cancer, ranking as the sixth most common cancer worldwide and the fourth leading cause of cancer-related deaths. Most HCC cases originate from cirrhotic livers, typically due to chronic liver diseases, such as hepatitis B (HBV) and hepatitis C (HCV) infections, and alcoholism. HCC cells often harbor numerous somatic mutations that are implicated in HCC development, but epigenetic factors, such as miRNA interference, can also affect HCC initiation and progress. miRNA-221 has been explored as a factor affecting HCC development in HCC of viral etiology, but little is known about its effects on gene expression in alcohol-associated HCC. This study aimed to explore potentially similar gene expression aberrations underlying viral and alcohol-induced HCC. We analyzed available transcriptome data from non-tumor hepatocytes and viral-induced HCC tissues. The most notable differences in gene expression associated with miRNA-221 between non-tumor hepatocytes and viral-induced HCC involved *NTF-3* and *MYBL1* genes. To assess these data in alcohol-induced HCC, we examined 111 tissue samples: tumor tissue and cirrhotic tissue samples from 37 HCC patients and 37 samples from non-tumor liver tissue using RT-Q PCR. We found no significant difference in *NTF-3* expression, but *MYBL1* expression was significantly lower in HCC tissue compared to non-tumor hepatocytes and cirrhotic tissue. Our findings highlight the importance of the *MYBL1* gene in HCC development and emphasize the need for diverse approaches in evaluating tumor mechanisms.

## 1. Introduction

Hepatocellular carcinoma (HCC) is the most common primary malignant epithelial neoplasm of the liver [1]. It is the sixth most common cancer in the world, with the highest incidence in Africa and Southeast Asia, and the fourth leading cause of cancer-related deaths, making it one of the leading health problems worldwide [1,2]. Between 80% and 90% of HCCs develop from a cirrhotic liver, which occurs as the end stage of chronic liver disease. Liver cirrhosis is one of the leading risk factors for the development of HCC [1,2,3]. Hepatitis B (HBV) and hepatitis C virus (HCV) infections and alcoholism are the most common risk factors for the development of chronic liver diseases and cirrhosis and are responsible for the development of more than 80% of all HCC cases [1,2,3,4,5,6,7,8].

Although HCC can arise as a result of various carcinogenic factors, it is now known that there are some common characteristics in the mechanism of HCC development from different etiologies, such as viral and alcoholic etiologies. Each risk factor for the development of HCC affects liver cells over a long period, leading to somatic and epigenetic mutations and changes in the damaged cells [9,10]. Between 60 and 70 somatic mutations can be detected in HCC cells [10]. There are approximately 34 mutations and gene changes that can be detected in all HCCs regardless of their etiology [10], and the highest similarity in gene mutations is between HCCs resulting from HBV infection and those resulting from excessive alcohol consumption [11]. Aside from mutations, epigenetic mechanisms, such as DNA methylation and histone modifications, and microRNA (miRNA) interference, can also affect HCC initiation and growth [12,13].

In the last decade, miRNAs have been recognized as important participants in tumor development. More than 250 miRNAs have been discovered in normal liver tissue, and one of the most significant miRNAs in the development of HCC is miRNA-221 [14,15,16,17,18,19]. Overexpressed miRNA-221 has been observed in HCC of different etiologies, especially in subtypes related to viral etiology, where its role and the genes it regulates are most studied [14,15,17,18,19,20,21]. The roles of miRNA-221-regulated genes are rarely studied in HCC of alcoholic etiology. As the tumorigenesis in HCC due to HBV infection has been shown to be similar to that caused by excessive alcohol consumption [10,11,22,23], this research aims to expand the understanding of the mechanism of HCC development and to analyze if the mechanisms of HCC development involve altered expression of the same genes regardless of its etiology. Exploring similarities between viral- and alcohol-induced HCC is crucial for uncovering shared molecular mechanisms that drive tumorigenesis. Identifying common pathways, such as those involving NTF-3 and MYBL1, could lead to the development of therapies that work for all HCCs independent of etiology, simplifying drug discovery. Since HCCs of all etiologies share factors like inflammation and liver damage, identifying common molecular drivers may also aid in preventing HCC progression and accelerating new treatment applications.

## 2. Results

### 2.1. Differentially Expressed Genes in HCC of Viral Etiology and Primary Hepatocytes

**Figure 1:**
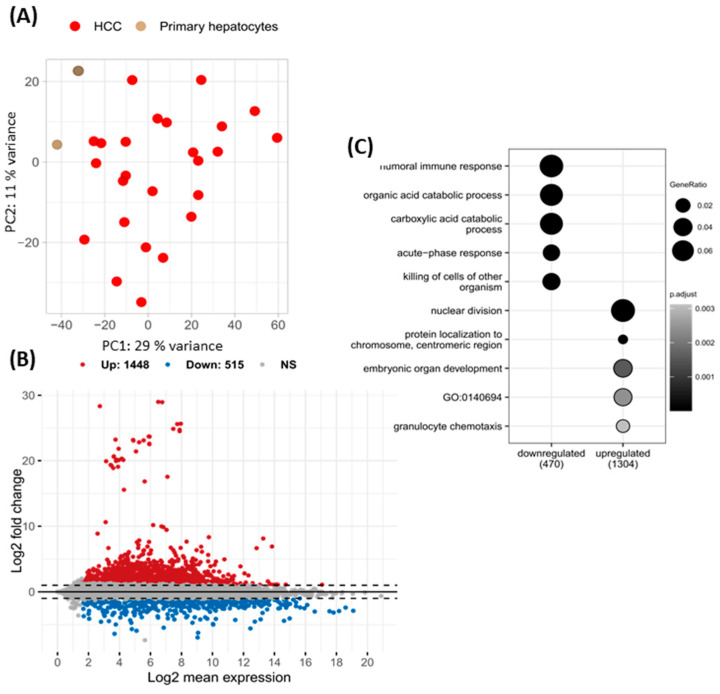
Differential expression analysis between hepatocellular carcinoma (HCC) tissues and primary hepatocytes. Principal component analysis (PCA) visualizing the overall gene expression variability between publicly available HCC and non-adjacent tumors from GSE105130, primary hepatocytes from EGA, and recurrent, tumor, and normal tissues from TCGA-LIHC cohort (**A**). PCA for only HCC patients with known etiology (risk factor) (**B**). MA plots illustrating differentially expressed genes between the HCC from GSE105130 and primary hepatocytes with groups expressed based on an absolute log2 fold change greater than 1 and a *p*-value less than 0.05, as determined by the Wald test using the DESeq2 package (**B**). Over-representation analysis of Gene Ontology (GO) terms in genes upregulated and downregulated in HCC from GSE105130 compared to primary hepatocytes (**C**).

To analyze differential gene expression between non-tumor primary hepatocytes, non-tumor liver tissue, and HCC tissue (GSE105130), publicly available datasets were used. Principal component analysis (PCA) clearly distinguished two separate clusters corresponding to each group (Figure 1A). The first two principal components (PC1 and PC2) explained 40% of the total variance. Additionally, we conducted a PCA on the selected HCC dataset with viral etiology group and TCGA-LIHC samples. The selected GEO samples were grouped with TCGA HCC samples, and no additional subclusters based on etiology were observed (Supplementary Figure S1). In total, 1448 upregulated and 515 downregulated differentially expressed genes (DEGs) were identified in HCC compared to primary hepatocytes (Figure 1B). Over-representation analysis of Gene Ontology (GO) terms revealed that the downregulated DEGs in HCC were predominantly involved in the humoral immune response, acute-phase response, and organic acid catabolic processes. In contrast, the upregulated DEGs were primarily associated with nuclear division (Figure 1C).

### 2.2. miRNA-Regulated Genes Characteristic for HCC

**Figure 2:**
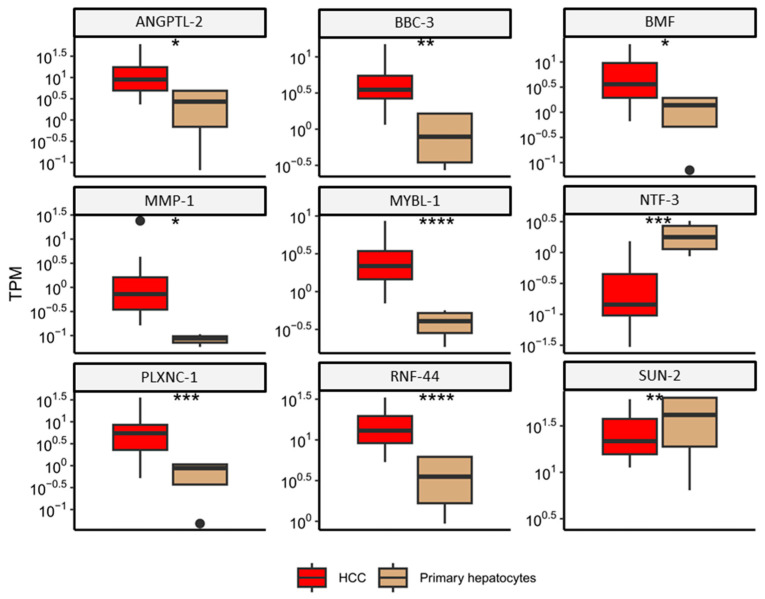
Differentially expressed genes regulated by miRNA-221 in hepatocellular carcinoma (HCC) patients compared to primary hepatocytes. Genes were identified as differentially expressed based on an absolute log2 fold change greater than 1 and a *p*-value less than 0.05, as determined by the Wald test using the DESeq2 package. ns: *p* > 0.05 *: *p* ≤ 0.05, **: *p* ≤ 0.01, ***: *p* ≤ 0.001, ****: *p* ≤ 0.0001. Angiopoietin-like protein 2 (*ANGPTL2*), bcl-2-binding component 3 (*BBC3*), bcl2 modifying factor *(BMF*), matrix metalloproteinase-1 (*MMP1*), MYB proto-oncogene like 1 (*MYBL1*), neurotrophin 3 (*NTF-3*), plexin C1 (*PLXNC1*), ring finger protein 44 (*RNF44*), and sad1 and UNC84 domain containing 2 (*SUN2*).

Based on a literature search, we compiled a list of differentially expressed genes in HCC of viral etiology that are regulated by miRNA. This list of genes for HCC of viral etiology, regulated by miRNA-221, was cross-referenced with detected differentially expressed genes (DEGs). Out of the 84 analyzed genes, nine were found to be differentially expressed in HCC compared to primary hepatocytes (angiopoietin-like protein 2 (*ANGPTL2*), bcl-2-binding component 3 (*BBC3*), bcl2 modifying factor (*BMF*), matrix metalloproteinase-1 (*MMP1*), MYB proto-oncogene like 1 (*MYBL1*), neurotrophin 3 (*NTF-3*), plexin C1 (*PLXNC1*), ring finger protein 44 (*RNF44*), and sad1 and UNC84 domain containing 2 (*SUN2*)) (Figure 2). Notably, *MYBL1* and *RNF44* were the most significantly upregulated genes in HCC, whereas *NTF-3* was the most significantly downregulated. For downstream analysis with qPCR and further evaluation in our study with alcohol-related HCC etiology, we selected the *NTF-3* and *MYBL1* genes.

### 2.3. NTF-3 and MYBL1 Expression in Samples from Patients with Alcohol-Associated HCC, Cirrhotic Tissue, and Non-Tumor Liver Tissue

**Figure 3:**
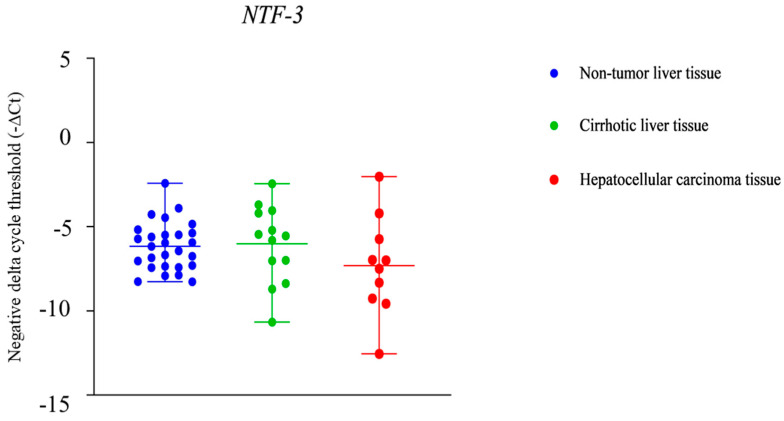
Difference in the neurotrophin 3 (*NTF-3*) gene expression levels between non-tumor liver tissue, cirrhotic liver tissue, and hepatocellular carcinoma (HCC) tissue.

The *NTF-3* gene showed no statistically significant difference in the expression levels between HCC tissue, cirrhotic liver tissue, and non-tumor liver tissue (Figure 3).

**Figure 4:**
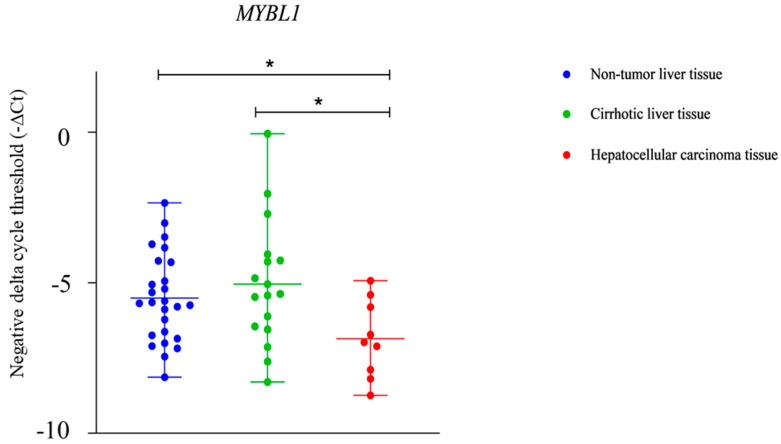
The results of this study have shown a significantly downregulated MYB proto-oncogene like 1 (*MYBL1*) in the hepatocellular carcinoma (HCC) tissue compared to the non-tumor liver tissue, as well as a significantly downregulated *MYBL1* gene expression in the HCC tissue compared to cirrhotic liver tissue (* *p* < 0.05).

**Figure 5:**
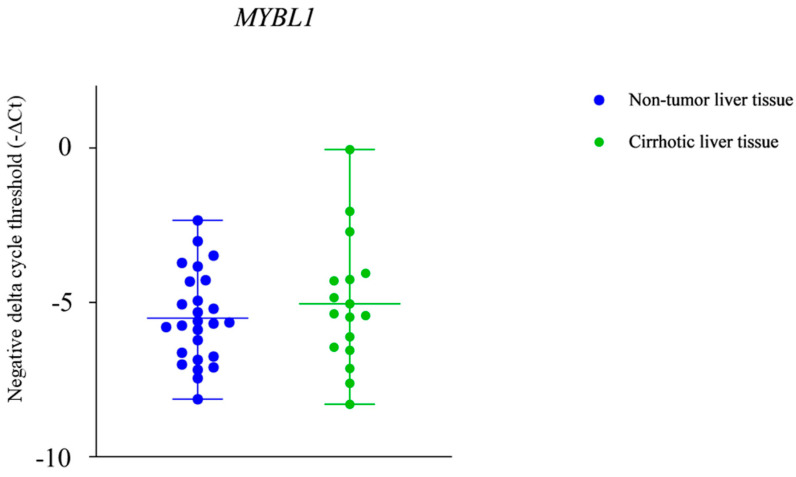
Statistically significant difference in the expression level of the MYB proto-oncogene like 1 (*MYBL1*) between the cirrhotic liver tissue and non-tumor liver tissue was not found.

The expression levels of the *MYBL1* gene showed a statistically significant difference between HCC tissue and both non-tumor liver tissue and cirrhotic liver tissue, but there was no statistically significant difference in expression between non-tumor liver tissue and cirrhotic liver tissue (*p* < 0.05) (Figure 4 and Figure 5).

There is a statistically significant positive correlation between the *NTF-3* and *MYBL1* expression in the group of patients with HCC (*p* < 0.01) (information about the correlation coefficients of laboratory parameters and gene expression in subjects with HCC is available in Table S1). No statistically significant correlation between the *NTF-3* and *MYBL1* expression in the group of patients with liver cirrhosis or in the non-tumor liver tissue was found (information about the correlation coefficients of laboratory parameters and gene expression in subjects with HCC and control group is available in Tables S1 and S2).

A statistically significant difference in the expression of both *NTF-3* and *MYBL1* with respect to age, gender, cell differentiation of carcinoma, lymph-vascular invasion, and laboratory parameters was not found.

## 3. Discussion

In this study, in silico analysis discovered 1448 upregulated and 515 downregulated differentially expressed genes in HCC of viral origin compared to primary hepatocytes. When these genes were cross-referenced with genes known to be regulated by the miRNA-221, which is believed to be a marker for viral HCC development, we detected nine genes of interest: *ANGPTL2*, *BBC3*, *BMF*, *MMP1*, *MYBL1*, *NTF-3*, *PLXNC1*, *RNF44*, and *SUN2*. These genes are involved in key processes in HCC, such as apoptosis, cell proliferation, angiogenesis, tissue remodeling, and metastasis. It is known that the deregulation of the expression of these genes in response to chronic viral infection and inflammation can create a microenvironment conducive to the initiation and progression of HCC [15,16,24,25,26,27,28,29].

Out of those nine genes, two were selected for further analysis using samples from patients with alcohol-induced HCC due to their expression being most significantly down- or upregulated in HCC of viral origin compared to primary hepatocytes. *NTF-3* was considered to have significantly lower expression in HCC tissue than in non-tumor hepatocytes, while the *MYBL1* gene was observed to be significantly overexpressed in tumor tissue when compared with non-tumor hepatocytes using in silico analysis. NTF-3 is a neurotrophic factor, and its key role is in the development of the nervous system, particularly the enteric nervous system [23,30,31]. By binding directly to the tropomyosin receptor kinase C (TrkC), NTF-3 induces signal transmission, activating the MAP or PI3K/AKT signaling pathways and inhibiting cell apoptosis [32,33]. The *MYBL1* gene is a proto-oncogene whose protein product functions as a transcription factor that binds MYBL1-binding sites (MBS) and is also known as A-MYB [34,35]. It plays a crucial role in regulating the development of breast tissue, nervous tissue, B-lymphocytes, and spermatogenesis [34,35,36,37,38,39]. MYB protein family participates in cell differentiation, proliferation, and death [34,35,36]. Although these genes are expressed in different organs, certain organs may express more than one member of this family [35].

The results of our in silico analyses are consistent with previous studies that showed downregulated expression of *NTF-3* in HCC tissue compared to primary hepatocytes. Through direct effect on p75NTR, NTF-3 leads to cell death. It was shown that this action is reduced in HCC due to the low level of NTF-3, leading to poorer survival [40,41]. On the other hand, the direct binding of NTF-3 to the TrkC receptor leads to signal transmission and activation of the PI3K/AKT and MAPK signaling pathways, preventing cell apoptosis [32,33]. Previous studies showed that the decreased expression level of NTF-3 in the HCC cells is associated with poorer prognosis [24,31,41], suggesting greater significance of the NTF-3 and p75NTR interaction in HCC compared to NTF-3 and TrkC interaction. Contrary to our in silico results, we did not observe a statistical difference in *NTF-3* expression between patients’ samples of HCC tissue of alcohol etiology, cirrhotic tissue, and non-tumor hepatocytes. This result could be based on the differences in analyzed patient cohorts—in silico analysis was performed using data from HCC of viral origin, while our patients were all diagnosed with HCC of alcohol etiology. This result could be associated with the results by Guo et al. [42] and Zhang et al. [43], whose bioinformatical analyses did not yield significantly changed expression of *NTF-3* in alcohol-induced HCC compared to non-tumor liver or prognosis significance of NTF-3. Conflicting results on the expression of NTF-3 have also been found in other tumor types. Breast tumors with upregulated NTF-3 expression, compared to breast tumors with lower NTF-3 levels, have a greater ability to metastasize to the brain, particularly to form a macro-metastasis [44]. The decreased levels of NTF-3 in breast tumor tissue in the brain are associated with reduced metastatic growth [45]. Upregulated NTF-3 was observed in other tumor cells, for example, in pancreatic carcinoma tissue compared to normal pancreatic tissue [45]. The expression of the NTF-3 has also been shown in lung squamous cell carcinoma tissue, but its role in the mechanism of lung carcinoma development is unclear [46,47]. As NTF-3 is known to regulate the PI3K/AKT signaling pathway by binding directly to the TrkC receptor [24,31,32,33,45,48], it is evident that it might have a role in the development of different malignant tumors, but also that its aberrations could be different depending on the type of tumor.

Even more interesting results were discovered for *MYBL1* gene expression. In the in silico analysis, we observed that the *MYBL1* expression was significantly higher in tissue samples from patients diagnosed with HCC of viral origin compared to primary hepatocytes (Figure 2). However, the results of our study conducted on patient tissue samples from alcohol-induced HCC have shown a significantly lower *MYBL1* expression in the HCC tissue compared to the non-tumor liver tissue, as well as a significantly lower *MYBL1* expression in the HCC tissue compared to the cirrhotic liver tissue (Figure 4). Additionally, we did not find any association between the expression level of the *MYBL1* and other tumor characteristics, such as tumor cell differentiation or lympho-vascular invasion. As MYBL1 is involved in the regulation of cell proliferation and differentiation through its interaction with cyclin A and cyclin E, especially in the G1/S phase of the cell cycle [35,49], it is not unexpected that any change in the level of the *MYBL1* expression can cause changes in cell proliferation and differentiation, which can as a consequence become uncontrolled [34]. In that line, our results gathered from the patients’ samples could be explained by possible interaction between miRNA-221 and *MYBL1* mRNA, as it was shown that high levels of miRNA-221 were associated with lower expression of MYBL1 in HCC cells [16,20,21].

Previously, upregulated *MYBL1* expression was found in HCC tissue compared to the liver tissue adjacent to the tumor [27,50], even though it did not have significantly changed expression according to several bioinformatical analyses [44,45]. Increased expression of MYBL1 in HCC was significantly associated with HCC cell proliferation, metastasis, and poorer overall survival through the possible interaction between MYBL1 and TWIS1 protein, whose expression was also found to be upregulated in HCC tissue [50]. MYBL1 is also known to influence increased vascularization in HCC tissue through the interaction with ANGPT2 [27]. Furthermore, MYBL1 has also been researched in other malignant tumors—its high expression was noted in breast tumors, salivary gland tumors, and pediatric glioma [51,52,53,54]. In breast carcinoma tissue with overexpression of the HER2 receptor, a high level of miRNA-221 was also found, and it was shown that it leads to reduced expression of *MYB* and *MYBL1* [55].

Interestingly, when we evaluated the possible correlation between *NTF-3* and *MYBL1* gene expression in tissue samples from patients with HCC of viral origin, cirrhotic tissue, and non-tumor liver tissue, we found a positive correlation only in the HCC group. Both *NTF-3* and *MYBL1* play critical roles in cellular processes such as proliferation, differentiation, and apoptosis, although in different contexts and mechanisms. NTF-3, primarily involved in the nervous system, interacts with receptors like TrkC and p75NTR to influence the PI3K/AKT and MAPK signaling pathways, impacting cell survival and differentiation. On the other hand, MYBL1, a transcription factor, regulates cell proliferation and differentiation through its interaction with cyclins and other transcription factors. Their potential link could be through the PI3K/AKT pathway, which is activated by NTF-3 via TrkC receptor binding and is also implicated in the regulation of *MYB* family genes, including *MYBL1* [14,15,21,24,27,30,32,33,35,39,50,51,52,53,54,56,57,58,59,60,61]. This pathway’s involvement in cell survival and proliferation suggests that disruptions in NTF-3 signaling could indirectly affect MYBL1′s regulatory functions, particularly in contexts like tumorigenesis, where both genes have shown altered expressions. Hence, understanding the interplay between NTF-3-mediated signaling and MYBL1 activity could provide insights into their collective impact on tumor development and progression. The mechanisms leading to the upregulated *NTF-3* and *MYBL1* expression in tumor tissue of one organ or downregulated expression in tumor tissue of another organ are still unknown. The different expression in tumor cells from different organ systems further complicates the understanding of the role of NTF-3 and MYBL1 in tumorigenesis. However, in HCC, it seems that NTF-3 and MYBL1 orchestrate a complex regulatory network impacting various cellular processes, including cell cycle control, apoptosis, and cell migration.

## 4. Materials and Methods

### 4.1. Bioinformatic Analysis

#### 4.1.1. Publicly Available Datasets

In order to analyze differential gene expression between non-tumor primary hepatocytes, non-tumor liver tissue, and HCC tissue, publicly available datasets were used for primary hepatocyte sets in the Gene Expression Omnibus (GEO) database under accession codes GSE43984 and ERR030887 in Sequence Read Archive, for normal and HCC tissues from 25 patients set GSE105130. Information about data sets is available in Table S3. For exploratory and cluster analysis, we downloaded the gene counts of the TCGA-LIHC cohort produced with the STAR mapper. The gene count data were obtained via the TCGABiolinks package in R (https://rdrr.io/bioc/TCGAbiolinks/, access date: 15 January 2024) [62]. Additionally, clinical information for the TCGA-LIHC cohort was sourced from Supplementary Table S1 of the study by Ally et al. [63].

To analyze differentially expressed genes characteristic of HCC that are regulated by miRNA-221, a gene list was constructed using a literature search and Qiagen RT2 Profiler PCR Array list (Table S4).

#### 4.1.2. Quality Control and Mapping

Quality control of downloaded raw reads was assessed by FASTQC (version v0.11.9) [64]. Raw reads were trimmed using a sliding window of 10 and a quality score below 25, adapters were removed, and reads shorter than 60 bp and average quality below 20 were filtered by Trimmomatic (version 0.32) [65]. Reads were mapped onto the human genome (hg38) using STAR (version 2.5.3a) [66] with the default settings. Mapped reads were counted over gene features annotated from Ensembl (GRCh38.105) using featureCounts v.2.0.0. [67].

#### 4.1.3. Principal Component Analysis (PCA)

Genes with fewer than 10 reads across all samples were removed. The reads were adjusted using variance stabilizing transformations before conducting principal component analysis (PCA). PCA was performed using the top 3000 most variable features. This process was carried out separately for combined counts from all datasets and for HCC samples with known etiologies.

#### 4.1.4. Differential Expression Analysis between HCC of Viral Etiology and Primary Hepatocytes

We selected HCC samples of viral etiology from GSE105130, along with primary hepatocytes, for differential expression analysis. The DESeq2 package (https://bioconductor.org/packages/release/bioc/html/DESeq2.html, 15 January 2024) [68] in R was used to compute statistical fold changes using LFC shrinkage (https://rdrr.io/bioc/DESeq2/man/lfcShrink.html, 15 January 2024) [69] in gene expression of protein-coding genes. Differentially expressed genes (DEGs) are defined as genes with a higher absolute value of the logarithmic fold change in expression between two groups of 2 and a lower adjusted *p*-value with Benjamini–Hochberg correction of 0.05 Transcripts Per Million (TPM) was calculated from raw counts and used for visual comparison of genes. All analyses were conducted in the R programming environment, version 4.3.3 [70].

### 4.2. HCC Patients’ Samples

#### 4.2.1. Materials

##### Tissue Specimens

A total of 111 tissue samples fixed in formalin and embedded in paraffin were included in this study and divided into 3 groups. The first group consisted of 37 tumor tissue samples from patients newly diagnosed with alcohol-associated HCC. The second group consisted of 37 tissue samples from the same patient, but tissue representing liver cirrhosis was selected. All patients were treated from January 2013 to December 2020 at the Merkur University Hospital, Zagreb, Croatia. The control group consisted of 37 samples of non-tumor liver tissue from patients diagnosed with nonhepatic diseases due to liver metastasis in the period from January 2020 to December 2022. The tissue samples were 10 μm thick and stored in tubes at room temperature until the isolation of RNA. The study included patients of both sexes, over 18 years old, with proven chronic alcohol consumption, excluding hepatitis B and C virus infection and metabolic syndrome, who had undergone liver transplantation and had not received any form of therapy for hepatocellular carcinoma before the liver transplantation. The study was approved by the Ethics Committee of the Clinical Hospital Merkur and the School of Medicine, University of Zagreb. General data of the patients diagnosed with HCC and liver cirrhosis are shown in Table S5, while the laboratory parameters are shown in Table S6. The general patient data of the control group are presented in Table S7, and their laboratory parameters are shown in Table S8.

#### 4.2.2. Methods

##### Ribonucleic Acid (RNA) Isolation, Reverse Transcription, qPCR

RNA isolation from paraffin-embedded tissue samples of HCC, liver cirrhosis, and non-tumor liver tissue was performed using the Quick-DNA/RNA™ FFPE Kit (Zymo Research, Tustin, CA, USA) following the manufacturer’s instructions. The isolated RNA was transcribed into cDNA using the PrimeScript Reverse Transcriptase kit (Takara, Kusatsu, Japan) according to the manufacturer’s instructions, using random hexamers as primers (Invitrogen, Waltham, MA, USA).

Reverse transcription was performed using the SimpliAmp instrument (Applied Biosystems, Waltham, MA, USA) according to the following protocol: 10 min at 30 °C, 60 min at 42 °C, and 15 min at 70 °C. The cDNA samples were stored at −20 °C until real-time polymerase chain reaction.

Quantitative polymerase chain reaction (qPCR) was performed according to the protocol using the PowerUp SYBR Green kit (Applied Biosystems, Waltham, MA, USA), specific primers for the genes of interest (Origene Technologies Inc., Rockville, MD, USA), and primers for the endogenous control (GAPDH) [71] (Table 1). qPCR run was performed on 7500 Fast Real-Time PCR System (Applied Biosystems, Waltham, MA, USA) for 40 cycles, according to the optimized reaction conditions: activation of uracil-DNA glycosylase at 50 °C for 2 min, activation of the HotStart DNA Taq Polymerase enzyme at 95 °C for 10 min; denaturation at 95 °C for 15 s; annealing at 57 °C for 15 s; and extension of the chain at 72 °C for one minute. The results were analyzed using the ddCt method of relative quantification using *GAPDH* as endogenous control.

#### 4.2.3. Statistical Analysis

Mann–Whitney U-test was used for the comparison of differences in laboratory data and gene expression between groups. Spearman’s rho correlation was used to assess the possible dependence between laboratory data and gene expression in each group of samples. Statistical analysis was performed using STATISTICA 13.0 (StatSoft Inc. Tulsa, OK, USA), and the significance level was set at *p* < 0.05.

## 5. Conclusions

This study underscores the need for further research to elucidate the specific mechanisms by which NTF-3 and MYBL1 alter the cell processes in HCC in order to reveal potential therapeutic targets for better management of the very common yet specific alcohol-induced HCC.

## Figures and Tables

**Table 1:** qPCR primer sequences.

Gene	Primer	Primer Sequence
*MYBL-1*	MYBL-1 F	5′-CGTGGAGGCAAACGCTGTGTTA-3′
	MYBL1 R	5′-GGTGGATTTGATAGGAGAAGCAG-3′
*NTF-3*	NTF-3 F	5′-CAAGCAGATGGTGGACGTTAAGG-3′
	NTF-3 R	5′-TCGCAGCAGTTCGGTGTCCATT-3′
*GAPDH*	GAPDH F	5′-TCAAGGCTGAGAACGGGAAG-3′
	GAPDH R	5′-CGCCCCACTTGATTTTGGAG-3′

F—forward primer, R—reverse primer, MYB proto-oncogene like 1 (*MYBL1*), neurotrophin 3 (*NTF-3*), glyceraldehyde 3-phosphate dehydrogenase (*GAPDH*).

## Data Availability

Raw data are available upon request.

## Supplementary Materials

The following supporting information can be downloaded at https://www.mdpi.com/article/10.3390/ijms251910558/s1.
