# Supplementary material for: Gene Expression Aberrations in Alcohol-Associated Hepatocellular Carcinoma
Source: Int J Mol Sci. 2024 Sep 30;25(19):10558. doi: 10.3390/ijms251910558 (PMC11476681; doi:10.3390/ijms251910558)

Supplementary Tables

Table S1. The correlation coefficients of laboratory parameters and genes in subjects with hepatocellular carcinoma.

	Correlation Coefficient§								
**Laboratory parameters and genes**	**Bilirubin**	**AST**	**ALT**	**GGT**	**ALP**	**Albumin**	**AFP**	**NTF-3**	**MYBL1**
**Bilirubin**	1,000	,268	-,007	-,205	**,395***	**-,429***	-,232	-,139	,117
**AST**	,268	1,000	**,577****	,158	**,346***	-,281	**-,414***	-,152	,200
**ALT**	-,007	**,577****	1,000	**,507****	,242	-,007	-,266	-,067	-,117
**GGT**	-,205	,158	**,507****	1,000	,295	,014	,025	,164	-,050
**ALP**	**,395***	**,346***	,242	,295	1,000	-,314	,009	,285	,150
**Albumin**	**-,429***	-,281	-,007	,014	-,314	1,000	,176	-,030	-,300
**AFP**	-,232	**-,414***	-,266	,025	,009	,176	1,000	,233	,690
**NTF-3**	-,139	-,152	-,067	,164	,285	-,030	,233	1,000	**1,000****
**MYBL1**	,117	,200	-,117	-,050	,150	-,300	,690	**1,000****	1,000

§Spearman's correlation coefficient; **p<0.01; *p<0.05.

Table S2. The correlation coefficients of laboratory parameters and genes in control group subjects.

	Correlation Coefficient§						
**Laboratorijski parametri i geni**	Bilirubin	AST	ALT	GGT	ALP	NTF-3	MYBL1
**Bilirubin**	1,000	-,114	-,267	-,031	-,158	-,002	-,252
**AST**	-,114	1,000	**,662****	,240	**,332***	,012	,002
**ALT**	-,267	**,662****	1,000	**,342***	**,430****	,009	,033
**GGT**	-,031	,240	**,342***	1,000	**,453****	,106	-,043
**ALP**	-,158	**,332***	**,430****	**,453****	1,000	-,013	-,005
**NTF-3**	-,002	,012	,009	,106	-,013	1,000	,245
**MYBL1**	-,252	,002	,033	-,043	-,005	,245	1,000

§Spearman's correlation coefficient; **p<0.01; *p<0.05.

Table S3. Publicly available datasets used in the study.

sample_id,group,subject,original_pair_samples,type,Column1,Column2,Column3,Column4
SRR656273,Primary hepatocytes,SRS389152Â ,GSE43984,single-read,,,,
ERR030887,Primary hepatocytes,ERR030887,ERR030887,paired-end,,,,
SRR651663,Primary hepatocytes,SRS382947,,paired-end,,,,
SRR651664,Primary hepatocytes,SRS382947,,paired-end,,,,

SRR6188339,HCC,P1,,paired-end,,tumor,GSM2819768,P1_tumor
SRR6188341,HCC,P2,,paired-end,,tumor,GSM2819770,P2_tumor
SRR6188343,HCC,P3,,paired-end,,tumor,GSM2819772,P3_tumor
SRR6188345,HCC,P4,,paired-end,,tumor,GSM2819774,P4_tumor
SRR6188347,HCC,P6,,paired-end,,tumor,GSM2819776,P6_tumor
SRR6188349,HCC,P7,,paired-end,,tumor,GSM2819778,P7_tumor
SRR6188351,HCC,P8,,paired-end,,tumor,GSM2819780,P8_tumor
SRR6188353,HCC,P9,,paired-end,,tumor,GSM2819782,P9_tumor
SRR6188355,HCC,P10,,paired-end,,tumor,GSM2819784,P10_tumor
SRR6188357,HCC,P11,,paired-end,,tumor,GSM2819786,P11_tumor
SRR6188359,HCC,P12,,paired-end,,tumor,GSM2819788,P12_tumor
SRR6188361,HCC,P13,,paired-end,,tumor,GSM2819790,P13_tumor
SRR6188364,HCC,P14,,paired-end,,tumor,GSM2819793,P14_tumor
SRR6188366,HCC,P15,,paired-end,,tumor,GSM2819795,P15_tumor
SRR6188368,HCC,P16,,paired-end,,tumor,GSM2819797,P16_tumor
SRR6188370,HCC,P17,,paired-end,,tumor,GSM2819799,P17_tumor
SRR6188372,HCC,P18,,paired-end,,tumor,GSM2819801,P18_tumor
SRR6188374,HCC,P19,,paired-end,,tumor,GSM2819803,P19_tumor
SRR6188376,HCC,P20,,paired-end,,tumor,GSM2819805,P20_tumor
SRR6188378,HCC,P21,,paired-end,,tumor,GSM2819807,P21_tumor
SRR6188380,HCC,P22,,paired-end,,tumor,GSM2819809,P22_tumor
SRR6188382,HCC,P23,,paired-end,,tumor,GSM2819811,P23_tumor
SRR6188384,HCC,P24,,paired-end,,tumor,GSM2819813,P24_tumor
SRR6188386,HCC,P25,,paired-end,,tumor,GSM2819815,P25_tumor
SRR6188389,HCC,P28,,paired-end,,tumor,GSM2819818,P28_tumor

Table S4. Genes regulated by miRNA-221.

no	gene	synonym
27113	*BBC3*	*PUMA*
10018	*BCL2L11*	
90427	*BMF*	
664	*BNIP3*	
1027	*CDKN1B*	*P27KIP1*
1028	*CDKN1C*	*p57Kip2*
58487	*CREBZF*	
54541	*DDIT4*	*REDD1*
27123	*DKK2*	
2099	*ESR1*	*Era*
2353	*FOS*	
2309	*FOXO3*	
3383	*ICAM1*	
3815	*KIT*	*CD117*
4312	*MMP1*	
4603	*MYBL1*	
5520	*PPP2R2A*	
5728	*PTEN*	
6648	*SOD2*	
29110	*TBK1*	
3267	*AGFG1*	
23452	*ANGPTL2*	
55608	*ANKRD10*	
378	*ARF4*	
648	*BMI1*	
55589	*BMP2K*	
54897	*CASZ1*	
865	*CBFB*	
961	*CD47*	
22856	*CHSY1*	
3491	*CCN1*	
10238	*DCAF7*	
54165	*DCUN1D1*	
54878	*DPP8*	
8669	*EIF3J*	
2114	*ETS2*	
2332	*FMR1*	
22862	*FNDC3A*	
3344	*FOXN2*	
79690	*GAL3ST4*	
2771	*GNAI2*	
2887	*GRB10*	
3020	*H3-3A*	

143279	HECTD2	
10527	IPO7	
55614	KIF16B	
5597	MAPK6	ERK3
1955	MEGF9	
90007	MIDN	
4302	MLLT6	
23164	MPRIP	
4801	NFYB	
51701	NLK	
4908	NTF3	
10605	PAIP1	
56137	PCDHA12	
5295	PIK3R1	p85-ALPHA
23228	PLCL2	
10154	PLXNC1	
5529	PPP2R5E	
5861	RAB1A	
6047	RNF4	
22838	RNF44	
222194	RSBN1L	
7095	SEC62	
6492	SIM1	
6500	SKP1	
51312	SLC25A37	
81539	SLC38A1	
6579	SLCO1A2	
8467	SMARCA5	
8723	SNX4	
8428	STK24	
25777	SUN2	
6938	TCF12	
9874	TLK1	
23023	TMCC1	
9760	TOX	
1831	TSC22D3	
7326	UBE2G1	
389856	USP27X	
7716	VEZF1	
22911	WDR47	
55884	WSB2	

Table S5. General data of the patients diagnosed with HCC and liver cirrhosis.

		N	%
**Sex**	Female<br>Male	2<br>35	5<br>95
**Age**	≤ 44 years<br>> 44 years	1<br>36	3<br>97
**Average age at the time of diagnosis**	62 years (range from 44 to 76 years)		
**Gradus**	Well-differentiated (G1 and G2)<br>Poorly differentiated (G3 and G4)	19<br>18	51<br>49
**Lymph-vascular invasion**	Yes<br>No	10<br>27	27<br>73

Table S6. Laboratory parameters of the patients diagnosed with HCC and liver cirrhosis.

	**Bilirubin**	**AST**	**ALT**	**GGT**	**ALP**	**Albumin**	**AFP**
**Reference rate**	3 – 20 µmol/L	11 – 34 U/L	8 – 41 U/L	9 – 35 U/L	64 – 153 U/L	41 – 51 g/L	< 7 µg/L
**Changed**<br>**Lower**<br>**Higher**	31<br>0<br>31	31<br>0<br>31	19<br>0<br>19	36<br>0<br>36	9<br>1<br>8	31<br>31<br>1	18<br>0<br>18
**Normal**	6	6	18	1	28	3	17
**No date**						2	2

Table S7. General data of the patients in control.

		N	%
**Sex**	Female<br>Male	20<br>17	54<br>46
**Age**	≤ 44 years<br>> 44 years	4<br>33	11<br>89
**Average age at the time of diagnosis**	63 years (range from 41 to 78 years)		

Table S8. Laboratory parameters of the patients in the control group.

	Bilirubin	AST	ALT	GGT	ALP
**Reference rate**	3 – 20 µmol/L	11 – 34 U/L	8 – 41 U/L	9 – 35 U/L	64 – 153 U/L
**Changed**<br>**Lower**<br>**Higher**	0<br>0<br>0	7<br>0<br>7	5<br>0<br>5	15<br>1<br>14	10<br>6<br>4
**Normal**	37	30	32	22	27
**No date**					

Figure S1. Principal component analysis on the selected viral HCC dataset and TCGA-LIHC samples.

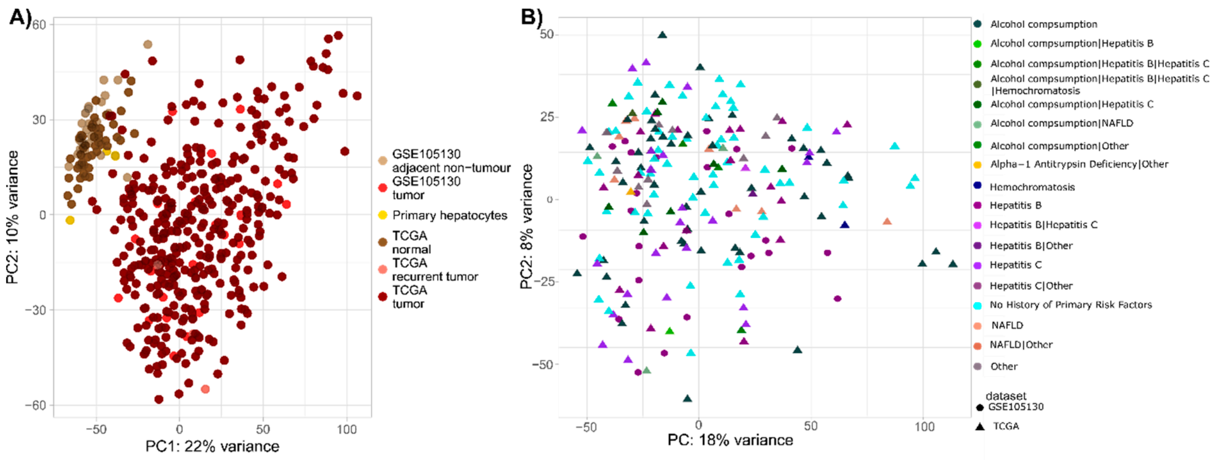

Supplement: Supplementary file 1 [file ijms-25-10558-s001.zip › ijms-3181987-supplementary.pdf]
